# Long non-coding RNA NKILA inhibits migration and invasion of tongue squamous cell carcinoma cells via suppressing epithelial-mesenchymal transition

**DOI:** 10.18632/oncotarget.11528

**Published:** 2016-08-23

**Authors:** Wei Huang, Xiuying Cui, Jianing Chen, Yuhuan Feng, Erwei Song, Jinsong Li, Yujie Liu

**Affiliations:** ^1^ Guangdong Provincial Key Laboratory of Malignant Tumor Epigenetics and Gene Regulation, Sun Yat-Sen Memorial Hospital, Sun Yat-Sen University, Guangzhou, China, 510120; ^2^ Breast Tumor Center, Sun Yat-Sen Memorial Hospital, Sun Yat-Sen University, Guangzhou, China, 510120; ^3^ Medical Research Center, Sun Yat-Sen Memorial Hospital, Sun Yat-Sen University, Guangzhou, China, 510120; ^4^ Department of Oral and Maxillofacial Surgery, Sun Yat-Sen Memorial Hospital, Sun Yat-sen University, Guangzhou, China, 510120

**Keywords:** long non-coding RNAs, NKILA, tongue squamous cell carcinoma, NF-κB, migration

## Abstract

Long non-coding RNAs (lncRNAs) have emerged recently as key regulators of tumor development and progression. Our previous study identified an NF-KappaB interacting lncRNA (NKILA) which was negatively correlated with breast cancer metastasis and patient prognosis. However, its clinical significance and potential role in Tongue squamous cell carcinoma (TSCC) remain unclear. Here we show that NKILA is down-regulated in TSCC cancer tissues than that in matched adjacent noncancerous tissues. And low NKILA expression in TSCC is significantly correlated with tumor metastasis and poor patient prognosis. *In vitro*, overexpression of NKILA decreases TSCC cells migration and invasion. Mechanistic study shows that NKILA inhibits the phosphorylation of IκBα and NF-κB activation as well as the induction of the epithelial-mesenchymal transition (EMT) process. Ectopic expression of NKILA in Tscca cells inhibits NF-κB activator TNF-α-promoted cell migration and invasion, while applying NF-κB inhibitor Bay-117082 or JSH-23 in NKILA silenced CAL27 cells reverses cell migration capacity to lower level. *In vivo* experimental metastasis model also demonstrates NKILA inhibits lung metastasis of NOD/SCID mice with TSCC tumors. These results suggested that NKILA is a vital determinant of TSCC migration and invasion and NF-κB signaling pathway mediates this effect. Given the above mentioned function of NKILA, it could act as a potential predictor for overall survival in patients with TSCC and a potential therapeutic target for TSCC intervention.

## INTRODUCTION

Tongue squamous cell carcinoma (TSCC) is the most common epithelial cancer identified in the oral cavity [1]. Despite the advancement of surgery, radiation and chemotherapy, the TSCC 5-year survival rate has not significantly improved during the past decades. It is mainly due to regional recurrence and lymph node metastasis [2]. However, the precise molecular mechanisms underlying TSCC metastasis and progression remain poorly understood. Therefore, it is vital to identify novel biomarkers and effective therapeutic strategies for TSCC.

Nuclear factor-κB (NF-κB) is a family of transcription factors that play critical roles in multiple physiological and pathological processes including inflammation, immunity, cell proliferation, differentiation, and survival [3]. Usually, NF-κB components are retained in the cytoplasm in an inactive state through association with IκB. Upon stimulation, activated IκB kinase complex (IKK) phosphorylates IκBα, and triggers its ubiquitination and subsequent degradation. As a consequence, NF-κB p65/p50 heterodimer is released and translocated to the nucleus, binding to its cognate DNA motifs, and regulating their expression [4, 5]. To date, extensive studies had shown that NF-κB is essential both for the induction and maintenance of Epithelial-mesenchymal transition (EMT) and for *in vivo* metastasis [6]. EMT is a common morphologic transformation in cancer cells that causes loss of cell-cell adhesion and increases cell motility which plays an important role in tumor progression and metastasis [7]. A serial of transcriptional repressors, such as Zeb-1/2, Twist1, Snail, and Slug control the EMT process by recruiting histone deacetylases to the E-box elements of E-cadherin promoter, leading to transcriptional silence of E-cadherin expression [8]. NF-κB was reported to binds to the promoters of the E-cadherin repressor genes and therefore regulates the EMT phenotype and migration.

It is well known that protein-coding genes account for < 2% of the total genome DNA, whereas a large number of the human genome can be transcribed into non-coding RNAs [9–11]. Among them are long non-coding RNAs (lncRNAs), which are more than 200nt in length with no protein-coding capacity [12]. To date, several lncRNAs had been identified to be dysregulated in a range of human cancers and contributing to tumorigenesis and tumor progression. Although the exact mechanisms by which lncRNAs function remain to be elucidated, accumulating studies suggested lncRNAs participated in biological regulation mainly due to their ability to interact with proteins, especially key signaling pathway proteins.

Our previous study found a nuclear transcription factor NF-κB interacting lncRNA (NKILA) which inhibited breast cancer cell migration by binding to NF-κB/IκB interface and directly blocking the phosphorylation site of IκB, and therefore inhibiting IKK-induced IκB phosphorylation and NF-κB activation [13]. However, the role of NKILA in TSCC is still elusive. In this study, we aimed to investigate the role of NKILA in regulating the NF-κB activity, tumor cell EMT and metastasis in TSCC, as well as the clinical significance of NKILA in predicting tumor metastasis and patient prognosis.

## RESULTS

### NKILA reduction predicts poor clinical outcome in patients with TSCC

**Figure 1:**
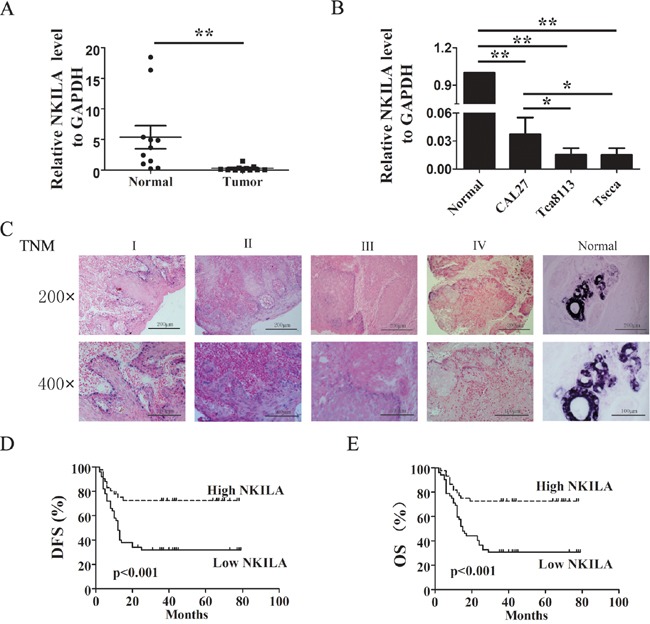
Relative NKILA expression in TSCC and its relationship with patients prognosis **A.** The expression levels of NKILA in TSCC tissues and adjacent noncancerous tissues, shown by qRT-PCR; **B.** NKILA expression levels in different ability of invasion of TSCC cell lines; **C.** Representative images of in situ hybridization for NKILA in paraffin-embedded TSCC tissue of different clinical stages and non-cancerous tissue. The magnificant is 200×(upper) and 400× (lower); **D.** & **E.** Kaplan–Meier survival curve of TSCC patients with low NKILA (SI<6) and high NKILA (SI≥6) levels, (D) Disease-free survival (DFS), (E) Overall survival (OS). Scale bars as indicated in the pictures. *p<0.05; **p<0.01.

Our previous report suggested the reduced NKILA is associated with clinical invasion and metastasis of breast cancer [13]. Here, we examined the expression pattern of NKILA in ten paired TSCC and matched adjacent noncancerous tissues. We found that, as shown in Figure 1A, NKILA was significant highly expressed in the adjacent noncancerous tissues than in TSCC tissues. In situ hybridization (ISH) in paraffin-embedded samples showed NKILA expression was decreased in the samples with high TNM stage (Figure 1C). Also in TSCC cell lines, the expression of NKILA was higher in freshly isolated normal tongue squamous epithelial cells or low-metastatic cell lines (CAL27) than in high-metastatic cell lines (Tca8113 or Tscca) (Figure 1B).

To further understand the significance of NKILA in TSCC, we analyzed the correlation between NKILA expression and the clinicopathological status of 96 patients with TSCC (Figure 1C and Table 1). Decreased NKILA expression level in TSCC was significantly correlated with tumor size (P=0.001, Table 1), advanced clinical staging (P=0.001, Table 1) and lymph node metastasis (P=0.001, Table 1). However, Statistical analyses showed no significant correlation between patient gender or age or histological classification with the expression levels of NKILA.

**Table 1:** Correlation between the clinicopathologic features and expression of NKILA. T, tumor; N, lymph node

Characteristics	NKILA in situ hybridization (%)		*P*
	No. of low expression	No. of high expression	
**Age (y)**			
<60	34 (51.5)	32 (48.5)	0.439
≥60	18 (60.0)	12 (40.0)	
**Gender**			
Female	19 (54.3)	16 (45.7)	0.986
Male	33 (54.1)	28 (45.9)	
**Histological classification**			
Well	4 (80.0)	1 (20.0)	0.075
Mediate	37 (59.7)	25 (40.3)	
Poor	11 (37.9)	18 (62.1)	
**Clinical stage**			
I-II	12 (32.4)	25 (67.6)	0.001
III-IV	40 (67.8)	19 (32.2)	
**T classification**			
T1-T2	22 (45.2)	33 (54.8)	0.001
T3-T4	30 (75.0)	11 (25.0)	
**N classification**			
N0	18 (36.0)	32 (64.0)	0.001
N1	18 (72.0)	7 (28.0)	
N2	16 (76.2)	5 (23.8)	
**Relapse**			
Yes	19 (38.8)	30 (61.2)	0.002
No	33 (70.2)	14 (29.8)	
**Status**			
Survival	17 (34.7)	32 (65.3)	0.000
Dead	35 (74.5)	12 (25.5)	

To determine relationship between NKILA expression and TSCC patients' prognosis, we evaluated the correlation between NKILA expression and patients' disease-free survival (DFS) and overall survival (OS) by Kaplan–Meier analysis. The results showed that 5 years of DFS for high NKILA expression was 22.7%, while was 9.6% for low NKILA expression. The median survival time for low NKILA expression was 12 months (Figure 1D, p<0.001). Moreover, 5 years of OS for high NKILA expression was 27.3%, while was 9.6% for low NKILA expression. The median survival time for low NKILA expression was 15 months (Figure 1E, p <0.001). To further assess whether NKILA expression can be identified as a prognostic predictor for TSCC patients, the univariate and multivariate survival analyses were performed. By univariate analysis, the variables clinical stage (P < 0.001), tumor size (P = 0.001) and NKILA expression level (P < 0.001) were found to be significantly associated with prognosis (Table 2). Multivariate analyses were used to determine whether NKILA expression level was an independent prognostic factor of clinical outcomes. According to our results, NKILA expression level showed significant prognostic effects on OS, independent of various clinical variables (Table 2).

**Table 2:** Univariate and ultivariate analysis of different prognostic variables in patients with TSCC using Cox regression analysis. T, tumor; SE, standard error

	Univariate analysis			Multivarite analysis	
	NO. patients	*p*	Regression coefficient (SE)	*p*	Relative risk (95%)
**Histological classification**		0.029	−0.590 (2.620)	0.763	0.913 (0.504-1.652)
Well	5				
Mediate	29				
Poor	62				
**Clinical stage**		0.000	1.333 (0.371)	0.310	1.750 (0.594-5.154)
I-II	37				
III-IV	59				
**T classification**		0.001	0.970 (0.295)	0.445	1.358 (0.622-2.963)
T1-T2	55				
T3-T4	41				
**NKILA**		0.000	−1.182 (0.335)	0.042	0.476 (0.236-0.975)
SI<6	50				
SI ≥6	46				

**Figure 2:**
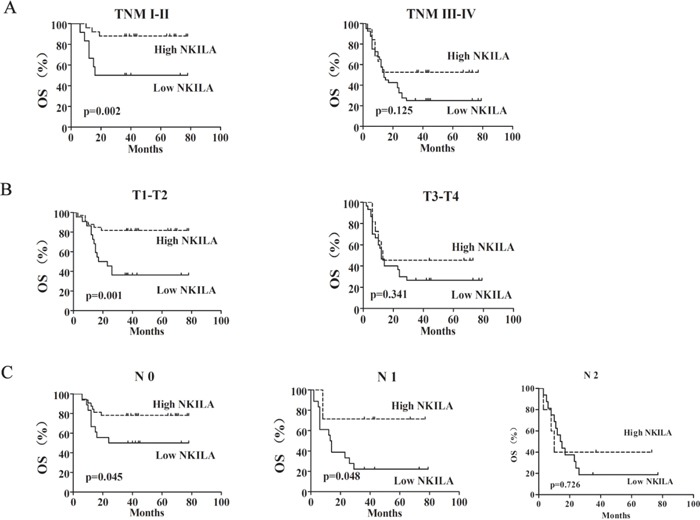
The expression of NKILA was associated with the progression of TSCC Kaplan-Meier analysis showing the overall survival (OS) of TSCC patients with low (SI<6) and high (SI ≥6) NKILA levels categorized according to TNM stage **A.**, tumor size **B.** and status of lymph nodes **C.**

Moreover, the prognostic value of NKILA expression in selective patient subgroups stratified by clinical stage, tumor size and lymph node status was analyzed. In light of our analysis, there was no difference between high and low NKILA expression groups in TSCC patients with a late stage (TNM III-IV, Figure 2A, right) or big tumor (T3-T4, Figure 2B, right) or lymph node metastasis (N2 classification, Figure 2C, right). However, patients with tumors exhibiting low NKILA expression had significantly shorter OS compared with those with high expression of NKILA in the clinical stage I-II (n=37, P= 0.002, Figure 2A, left), or the T1-T2 subgroup (n=55, P=0.001, Figure 2B, left) or N0 and N1 subgroup (n=50, P= 0.045 and n=25, P=0.048, Figure 2C). Taken together, these data indicated that down-regulation of NKILA was significantly associated with the progression of TSCC and predicted a poor prognosis.

### NKILA regulates the migration and invasion of TSCC cells

**Figure 3:**
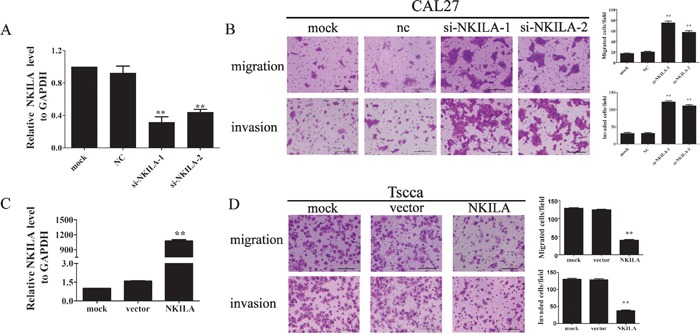
NKILA negatively regulated TSCC cell migration and invasion **A.** The knockdown efficiency of two specific siRNA against NKILA was examined by qRT-PCR in CAL27 cells; **B.** Silencing NKILA in CAL27 cells increased cell migration and invasion. The migrated or invaded cells were detected 12hrs or 24hrs after cells were seeded, respectively. Left panel was representative images and right panel was statistical column diagram; **C.** Analysis of NKILA expression levels in Tscca cells transfected with pcDNA3.1-NKILA (NKILA) or empty vector (vector) by qRT-PCR; **D.** Upregulation of NKILA in Tscca cells suppressed cell migration and invasion. The migrated or invaded cells were detected 3 hrs or 8 hrs after cells were seeded, respectively. Left panel was representative images and right panel was statistical column diagram. Scale bars as indicated in the pictures. *P<0.05, **p<0.01 versus corresponding controls.

As our clinical data indicated that NKILA expression levels negatively correlated with TSCC metastasis. We further investigated the function of NKILA in migration and invasion in TSCC cell lines. The low-metastatic cell line CAL27 which has the higher NKILA expression was transiently transfected with two specific NKILA siRNAs (si-NKILA-1 and si-NKILA-2). Both siRNAs efficiently silenced endougenous expression of NKILA (Figure 3A). CAL27 cells with down-regulated NKILA expression showed increased ability of migration and invasion than that in mock or negative control (NC) treated cells (Figure 3B). Also, ectopic expression of NKILA in high-metastatic TSCC cell line Tscca by transfecting with NKILA containing pcDNA3.1 (NKILA) markedly increaed the NKILA level than that in pcDNA3.1 vector transfection cells (vector) or mock group (Figure 3C). Elevated NKILA suppressed cell migration and invasion when compared to mock or vector group cells (Figure 3D). These data suggested that NKILA inhibits TSCC cells migration and invasion.

### NKILA inhibits IκB phosphorylation and NF-κB activation in TSCC cells

**Figure 4:**
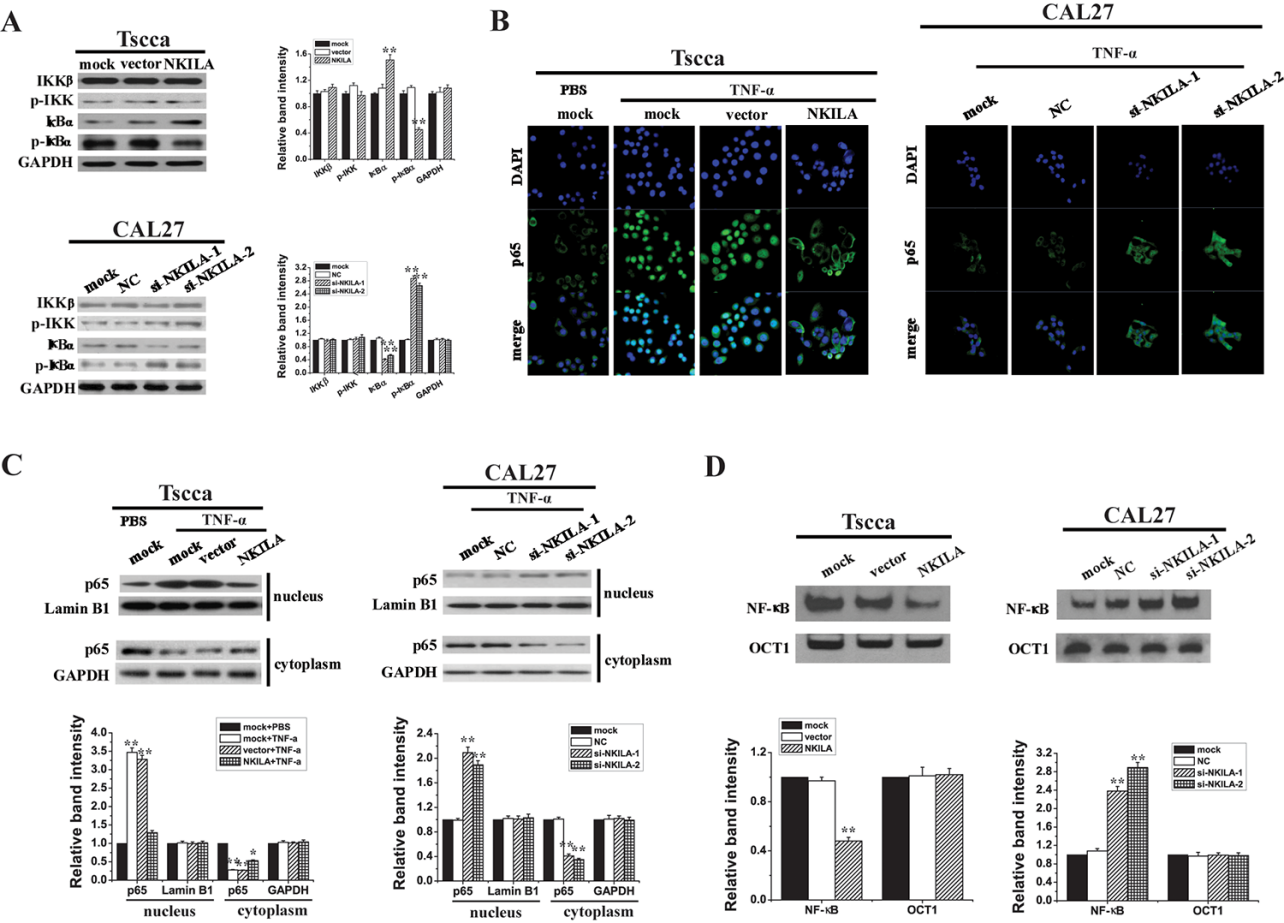
NKILA inhibits IκB phosphorylation and the activation of NF-κB in TSCC cells **A.** Western blotting showing total and phosphorylated IKK and IκBa in Tscca cells and CAL27 cells. Left panel was representative images and right panel was statistical column diagram; **B.** p65 nuclear translocation in Tscca expressing NKILA or CAL27 cells expressing NKILA siRNAs treated with TNF-α 20 ng/ml for 30 min or 5 ng/ml for 24h, respectively, assayed by immunofluorescent confocal microscopy; **C.** Western blotting for nuclear and cytoplasm p65 in Tscca cells and CAL27 cells. GAPDH or Lamin B is the loading control for cytoplasm or nuclear, respectively. Upper panel was representative images and lower panel was statistical column diagram; **D.** NF-κB activities of Tscca cells or CAL27 cells, assayed by EMSA. Upper panel was representative images and lower panel was statistical column diagram. *P<0.05, **p<0.01 versus corresponding mock group.

Because increased NF-κB activity is associated with enhanced invasiveness of tumor cells, we further detected the function of NKILA in NF-κB activation. Our results showed overexpression of NKILA in Tscca cells inhibited the phosphorylation of IκBα compared to that in mock or vector treated cells (Figure 4A). However, IκBα phosphorylation was significantly enhanced in CAL27 cells with suppressed expression of NKILA (Figure 4A). In both situations the phosphorylation of IKK was not changed obviously. These results suggested NKILA mainly affected IκBα phosphorylation and not influenced IKK activation. Next, we investigated the NF-κB activity by observing the nuclear translocation of the p65 subunit of NF-κB. We used a classical NF-κB inducer TNF-α to treat Tscca cells for 5min and found TNF-α promoted the p65 nucleus translocation, while overexpression of NKILA totally prevented this translocation with most of the p65 staining in cytoplasm around nuclear. On the other hand, silencing NKILA in CAL27 cells promoted the TNF-α induced nuclear translocation of p65 (Figure 4B). Additionally, Western blot showed similar results with immunofluorescence that overexpression of NKILA in Tscca cells increased the proportionate amount of p65 in the cytoplasm when treated with TNF-a for 5 minutes (Figure 4C). Conversely, silencing NKILA in CAL27 cells significantly increased the translocation of p65 from the cytoplasm to the nucleus when treated with TNF-α for 24 hours (Figure 4C). Also, we detected the NF-κB activity by using EMSA with canonical NF-κB probe which was a sequence located in the NF-κB/IκB interface. As shown in Figure 4D, knockdown of NKILA in CAL27 cells increased the binding of NF-κB with probe which means NF-κB are at activated form without the binding of IκB. And overexpression of NKILA in Tscca cells decreased NF-κB activities.

### NF-κB/Twist signaling pathway mediates NKILA regulated EMT and migration in TSCC cells

**Figure 5:**
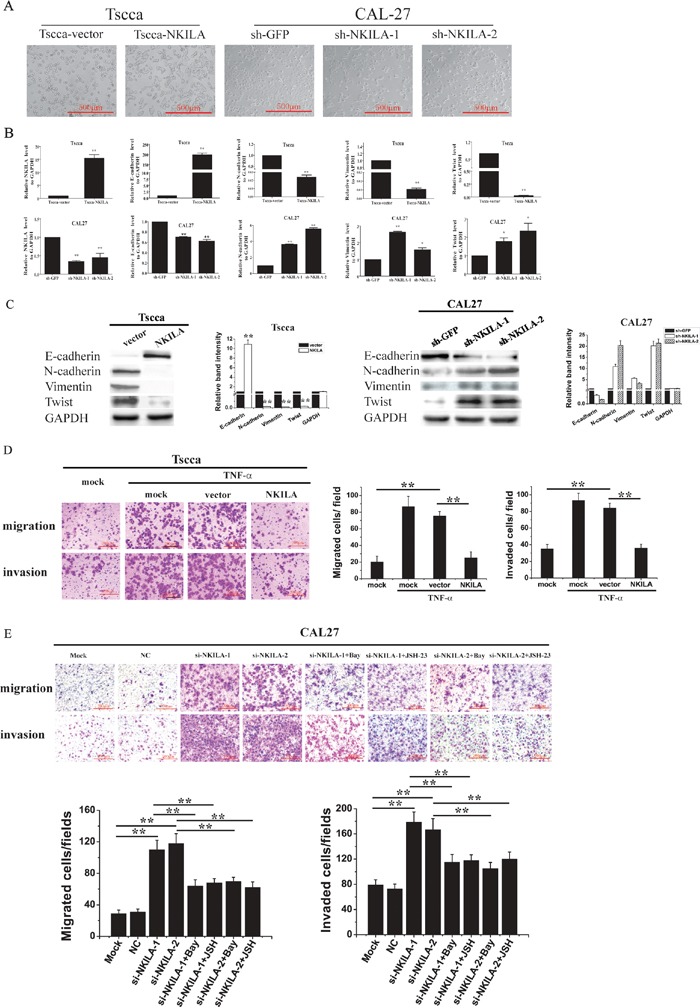
NKILA inhibits EMT and migration of TSCC cells via NF-κB /Twist signaling **A.** NKILA controls the EMT phenotype of TSCC cells, shown as bright field images; **B.** The levels of NKILA, Twist, E-cadherin, N-cadherin and Vimentin mRNA in Tscca cells and CAL27 cells, detected by qRT-PCR; **C.** The levels of NKILA, Twist, E-cadherin, N-cadherin and Vimentin protein in Tscca cells and CAL27 cells, examined by Western blot. Left panel was representative images and right panel was statistical column diagram. *P<0.05, **p<0.01 versus corresponding controls; **D.** migration and invasion of Tscca cells transiently transfected with mock, empty vector or NKILA in the presence or absence of 20 ng/ml TNF-α for 30 min. Left panel was representative images and right panel was statistical column diagram; **E.** migration and invasion of CAL27 cells transiently transfected with mock, NC or si-NKILAs in the presence or absence of 2 μM Bay-117082 or 30 μM JSH-23. Left panel was representative images and right panel was statistical column diagram. Scale bars as indicated in the pictures. *P<0.05, **p<0.01.

It was reported that NF-κB play pivotal roles in regulating tumor cell EMT. Since NKILA inhibits NF-κB activity, we hypothesized NKILA could influence TSCC cell EMT process. Our results showed Tscca cells stably expressing NKILA grew in clusters with tight cell-cell junctions, while the vector transfected cells separated from one another. In the case of CAL27 cells, the parent cells stably knockdown of GFP grew in cluster and had round shapes with tight cell-cell junctions, while NKILA deficiency cells displayed a spindle shape and separated from one another (Figure 5A). These results indicated silence of NKILA was related to the mesenchymal phenotype of cells. Then we detected the expression of EMT markers by qRT-PCR. The results showed that stably transfected with NKILA in Tscca cells reduced the expression of Twist (Figure 5B), while transduced with small hairpin RNA against NKILA (sh-NKILA-1 and sh-NKILA-2) increased Twist level. These results indicated NKILA might involve in regulating the EMT in TSCC cells. Then we detected the mRNA level of classical EMT markers E-cadherin, N-cadherin and vimentin. The results showed that the expression of E-cadherin mRNA was dramatically elevated accompanied by the expression of N-cadherin and vimentin significantly decreased in NKILA overexpressed Tscca cells. And the expression of E-cadherin mRNA was moderately decreased accompanied by the expression of vimentin and N-cadherin mRNA elevated in NKILA silenced CAL27 cells (Figure 5B). We also performed Western blot to examine the expression of EMT markers. Consistent with the qRT-PCR results, the Western blot showed significantly increase of E-cadherin and decrease of twist, vimentin and N-cadherin in NKILA overexpressed Tscca cells (Figure 5C). Meanwhile, decrease of E-cadherin and increase of twist, vimentin and N-cadherin were observed in NKILA silenced CAL27 cells (Figure 5C). These results showed NKILA regulated the EMT process of TSCC cells.

We then investigated whether NF-κB /Twist pathway is responsible for NKILA regulated cell migration. TNF-α, the classical NF-κB activator, promoted the migration and invasion of Tscca cells, which could be completely inhibited by simultaneous transfection of NKILA (Figure 5D). Knockdown of NKILA in CAL27 cells enhanced cell migration and invasion. Pretreatment of the NKILA deficiency cells with Bay-117082 or JSH-23, two inhibitors for NF-κB nuclear translocation, antagonized the effect of NKILA siRNA (Figure 5E), suggesting that NKILA exerts its effect via inhibiting NF-κB.

### NKILA suppresses TSCC tumor metastasis

**Figure 6:**
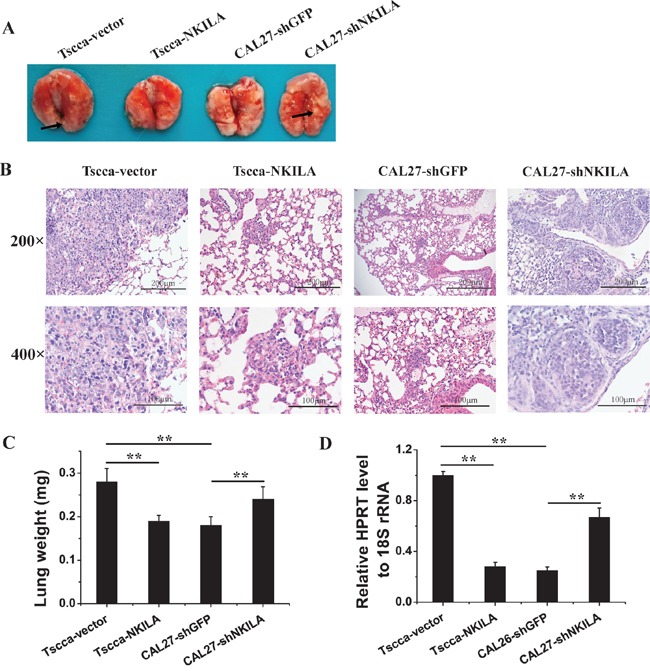
NKILA negatively regulates TSCC cells metastasis in NOD/SCID mice **A.** Lung images of female NOD/SCID mice intravenously injected with Tscca-vector, Tscca-NKILA, CAL26-shGFP, CAL27-shNKILA cells. (n=6 per group); **B.** representative microphotos of lung tissue sections stained with H&E. The magnificant is 200×(upper) and 400× (lower). Scale bars as indicated in the pictures; **C.** Wet lung weight in tumor-bearing mice; **D.** qRT-PCR for human HPRT mRNA normalized to mice 18S rRNA in the lungs of mice intravenously injected with Tscca or CAL27 cells. **p<0.01.

To further verify the role of NKILA in regulating TSCC migration and invasion, we injected 1×10^6^ Tscca cells stably expressing empty vector (Tscca-vector) or NKILA (Tscca-NKILA), or CAL27 cells stably transfected with sh-GFP (CAL27-shGFP) or sh-NKILA (CAL27-shNKILA) into the tail vein of NOD/SCID mice to produce experimental metastasis models. 8 weeks after tumor inoculation, the mice were sacrificed and lung and liver were observed the tumor metastasis. All the mice didn't develop liver metastasis. For the experimental lung metastasis, we could observe tumor nodules on the surface of lungs in Tscca control mice and CAL27-shNKILA mice. However, there was no visible metastasis in the lungs of Tscca-NKILA and CAL27-shGFP mice (Figure 6A). The extent of Tscca-NKILA and CAL27-shGFP cells metastasis was also reduced when assessed by hematoxylin and eosin (H&E) staining of lung tissue sections which we only observed micrometastasis (Figure 6B). The ratio of human HPRT mRNA relative to mouse 18S rRNA and the average wet lung weight also showed the extent of lung metastasis was negatively related to the expression level of NKILA (Figure 6C & 6D).

## DISCUSSION

LncRNAs are mRNA-like transcripts ranging in length from 200nt to ~100 kilobases (kb) that lack coding protein function [14]. In the past decades, accumulating evidence have indicated that lncRNAs were involved in a wide range of biological functions, such as cell proliferation, cell apoptosis and cell metastasis. Although lots of studies have demonstrated that lncRNAs deregulation is pivotal in cancer initiation and progression and metastatic spread, their biological functions and mechanisms of action are still not fully understood.

Recently, several lncRNAs have been documented to promote tumor progression and metastasis via different mechanisms. HOTAIR recruits polycomb repressive complex (PRC2) to specific targets genes promoter regions, leading to H3K27 trimethylation and epigenetic silencing of metastasis suppressor genes, and to increasing breast cancer invasiveness and metastasis [15]. H19 was overexpressed in many types of cancer including bladder, pancreatic and breast cancer. H19 increases bladder cancer metastasis by associating with EZH2, which increases Wnt/β-catenin activation and results in a decreased expression of E-cadherin [16]. H19 also promoted pancreatic cancer cell invasion and migration at least partially by derepressing let-7's inhibition on its target gene HMGA2 which induces EMT [17]. In Hepatocellular Carcinoma, lncRNA-activated by TGF-β (lncRNA-ATB) upregulated ZEB1 and ZEB2 by competitively binding the miR-200 family and then induced EMT and invasion. lncRNA-ATB could also bind IL-11 mRNA and increased IL-11 mRNA stability, causing autocrine induction of IL-11, and then activating STAT3 signaling [18]. The lncRNA Low Expression in Tumor (lncRNA-LET) was downregulated in hepatocellular carcinomas, colorectal cancers and squamous-cell lung carcinomas and inhibited the metastasis of hepatocellular carcinomas by stabilizing nuclear factor 90, which leads to hypoxia-induced cancer cell invasion [19].

Our previous study reported a novel mechanism of action for lncRNA that lncRNA NKILA could directly interact with signaling protein NF-κB by blocking the IKK phosphorylating sites of IκB and therefore inhibiting NF-κB mediated breast cancer cell apoptosis and migration. In this study, we also found IκBα phosphorylation was inhibited in Tscca cells by enforcing NKILA expression, but was enhanced in CAL27 cells by silencing NKILA. However, basal IKK phosphorylation was not affected by altering NKILA in both cell lines. Our previous study in breast cancer showed IKK kinase inhibitor could abolish the effects of NKILA-shRNA on IκB phosphorylation and NF-κB activation, indicating IKK is the upstream signal protein of NKILA. Furthermore, in cells with silenced IκB or trans-dominant IκB, knockdown of NKILA had no effects on NF-κB activation. Taken together, these previous study and our present results suggest NKILA blocks NF-κB activation by inhibiting IKK-induced IκB phosphorylation, but not affecting IKK activity (Figure 4).

Activated NF-κB translocates to the nucleus and binds to its cognate DNA motifs in the promoter regions and induces a diverse of genes expression which involved in multiple biological processes including migration and EMT. In this study, we found NKILA expression levels were higher in the low- metastatic TSCC cell lines (CAL27) than in the high-metastatic cell lines (Tscca and Tca8113). Enhancing NKILA expression in Tscca cells led to the significant inhibition of cell metastasis both at basal condition and TNF-α treatment condition (Figure 3D & 5D). Knockdown of NKILA expression in CAL27 cells enhanced migration and invasion which could be antagonized when applying NF-κB inhibitors (Figure 3B & 5E). These results suggested NF-κB mediated NKILA regulated TSCC cells mobility.

Twist is a basic helix-loop-helix (b-HLH) transcription factor that plays an essential role in mediating cell motility and invasiveness by regulating EMT [20]. Upregulation of Twist was associated with tumor aggressiveness and poor survival in several human cancers, such as hepatocellular cancer, breast cancer, pancreatic cancer, and gastric cancer [21–23]. Studies had reported in various cancer types that NF-κB regulated the expression of transcriptional repressor Twist and promoted cell EMT and metastasis [24–26]. In lung cancer, knockdown of BRMS1 drove NSCLC cells EMT and lung metastasis via NF-κB/Twist signaling pathway, while silencing of Twist not only blocked BRMS1^KD^ cells to undergo EMT and invasion, but also reversed the EMT process [27]. Consistent with the observation in lung cancer, Li et al found in breast cancer that NF-κB bond to the functional site in the Twist promoter and induced transcriptional upregulation of Twist as well as EMT and migration of cancer cells. Suppressing Twist abrogated NF-κB induced EMT, migration and invasion [28]. These results indicated Twist was required for NF-κB induced EMT and metastasis. In this study, we also found when overexpression of NKILA in Tscca cells which decreased the NF-κB activation, the expression of Twist was attenuated accompanied by the increase of E-cadherin and decrease of vimentin and N-cadherin, as well as the poor motility of the cells. On the contrary, knockdown of NKILA in CAL27 cells which promoted the translocation of p65 enhanced the expression of Twist, vimentin and N-cadherin, and the migration and invasion of cells. These results indicated Twist was the downstream signaling protein of NF-κB regulating TSCC cells EMT and migration.

Consistent with the *in vitro* study, NKILA negatively control the tumor metastasis in experimental metastasis model. Tscca-vector cells and CAL27-shNKILA cells were more prone to develop lung metastasis with H&E staining of large amount of tumor cells, while Tscca-NKILA and CAL27-shGFP cells were less efficient to form metastasis. H&E staining showed there had micrometastasis in lung sections. Notably, there has no liver metastasis observed (data not shown), probably because the liver metastasis are not easy to occur at TSCC cells experimental metastasis model.

Our experimental data strongly suggested NKILA play an important role in regulating TSCC cells migration and invasion. And the clinical data further proved the clinical significance of NKILA in TSCC. The results demonstrated that the expression of NKILA was significantly downregulated in TSCC tissues when compared to matched normal tissue. This expression pattern of NKILA in TSCC was consistent with our previous observation in breast cancer that NKILA expression in normal breast tissues was significantly higher than that in the ductal carcinomas in-situ without metastasis or metastasis. In TSCC the low expression level of NKILA was associated with tumor size, advanced clinical staging, lymph node metastasis and poor survival, indicating that the low NKILA expression may promote an aggressive phenotype of TSCC and serve as a prognostic marker.

**Figure 7:**
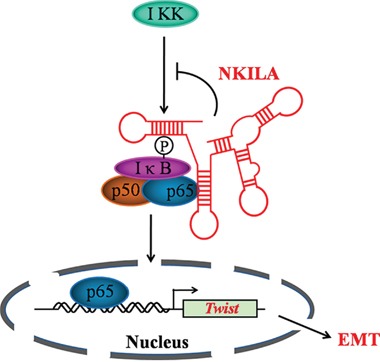
Schematic summary of the model of mechanism of NKILA

In summary, our present findings further proves that NKILA plays vital roles in controlling tumor cell migration and invasion by blocking the phosporylation of IκB, NF-κB activation and the following EMT process (Figure 7). Our findings indicate NKILA might be developed to a useful tool agent to inhibit NF-κB signaling pathway. Also, given the good prognostic value of NKILA for TSCC and migration inhibitory effects *in vitro*, NKILA also serves as a potential therapeutic target for TSCC treatment.

## MATERIALS AND METHODS

### Ethic statement

All samples were collected with informed consent from patients, and all related procedures of investigation have been conducted in accordance with the ethical standards and according to the Declaration of Helsinki and according to national and international guidelines and have been approved by the review board of Sun Yat-Sen Memorial Hospital at Sun Yat-Sen University.

### Patients and tissue specimens

A total of 96 paraffin-embedded TSCC samples from Sun Yat-Sen Memorial Hospital from 2008 to 2011 were examined in the present study. Fresh tumor samples of primary TSCC and matched adjacent non-TSCC tissues were obtained from Department of Oral & Maxillofacial Surgery of Sun Yat-Sen Memorial Hospital. The disease stages of the patients were classified according to American Joint Committee on Cancer (AJCC).

### Cell cultures and treatment

CAL27 was purchased from American Type Culture Collection (ATCC, Manassas, VA, USA), Tca8113 cell line was from Shanghai Jiao Tong University College of Stomatology. And Tscca was from School of Stomatology Wuhan University. The primary normal oral epithelial cells were isolated as previously reported [29]. Cells were cultured in Dulbecco's Modified Eagle's Medium (DMEM; Life Technologies, Carlsbad, CA, USA) supplemented with 10% fetal bovine serum (FBS; Life Technologies, USA). All the cells were maintained in a humidified atmosphere containing 5% CO_2_ at 37°C.

Transient transfection in TSCC cells was performed by using lipofectamine 2000 (Life Technologies, USA) according to the manufacture's protocol. And permanent transfection was conducted by using commercial Lentiviral supernatant (GenePharm, Shanghai, China) as previously reported [30].

### Quantitative real time PCR (qRT-PCR)

Total RNA from cancerous, noncancerous specimens or cell lines was extracted with the Trizol reagent (Life Technologies, USA) according to the manufacturer's protocol. The complementary DNA (cDNA) was synthesized using the PrimeScript RT reagent Kit (TaKaRa, Dalian, China). And the RNA expression levels were performed using the Roche LightCycler® 480 Real-Time PCR System. The gene-specific primers were as follows: GAPDH (sense: 5- ATC ACC ATC TTC CAG GAG CGA-3′; antisense: 5′- CCT TCT CCA TGG TGG TGA AGA C-3′); NKILA(sense: 5′-AAC CAA ACC TAC CCA CAA CG-3′; antisense: 5′-ACC ACT AAG TCA ATC CCA GGT G-3′); Twist (sense: 5′- GGA GTC CGC AGT CTT ACG AG-3′; antisense: 5′- TCT GGA GGA CCT GGT AGA GG-3′); E-cadherin (sense: 5′- GGA GGA GAG CGG TGG TCA AA-3′; antisense: 5′- TGT GCA GCT GGC TCA AGT CAA-3′); vimentin (sense: 5′- GAC AAT GCG TCT CTG GCA CGT CTT-3′; antisense: 5′- TCC TCCG CCT CCT GCA GGT TCT T-3′); N-cadherin (sense: 5′-CAC TGC TCA GGA CCC AGA T-3′; antisense: 5′-TAA GCC GAG TGA TGG TCC-3′); human HPRT (sense:5′- TTC CTT GGT CAG GCA GTA TAA TCC-3′; antisense: 5′- AGT CTG GCT TAT ATC CAA CAC TTC G-3′), mouse 18S rRNA (sense: 5′-CGG CTA CCA CAT CCA AGG AA-3′; antisense: 5′- GCT GGA ATT ACC GCG GCT-3′).

### Western blot analysis and subcellular fraction

Whole cell lysates were resolved by 10% SDS polyacrylamide gels electrophoresis (BioRad, Berkeley, CA, USA) and transferred to PVDF membranes. The membrane was probed with specific antibodies against human GAPDH (Sigma, USA), p65 (CST, Danver, MA, USA), IKK (CST, USA), p-IKK (CST, USA), IκB (CST, USA), p-IκB (CST, USA), E-cadherin (CST, USA), vimentin (CST, USA), N-cadherin (R&D, Minneapolis, MN, USA) or Twist (CST, USA). Peroxidase-conjugated anti-mouse IgG or anti-rabbit IgG (CST, USA) was used as secondary antibody. And the antigen-antibody reaction was visualized by using enhanced chemiluminescence assay (ECL, Thermo, Rockford, USA).

Subcellular fractions were prepared from TSCC cells with an NE-PER Nuclear and Cytoplasmic Extraction kit (Thermo Scientific, USA) according to manufacturer's instructions.

### Transwell assay

TSCC cells were transiently transfected with NKILA siRNAs and NKILA overexpression plasmid, as well as their corresponding contols. After incubation for 48 hours, the cells were digested and 1×10^5^ cells in serum-free DMEM were seeded on the upper inserts of 24-well Boyden chambers (Corning, New York, NY, USA) with (for invasion) or without (for migration) matrigel (R&D, USA). DMEM medium supplemented with 10% FBS was added to the lower chambers. For Tscca cells, we detected cell mobility after 3 hrs (for migration) or 8 hrs (for invasion) culture. And for CAL27 cells, we detected cell mobility after 12 hrs (for migration) or 24 hrs (for invasion) culture. The migrated and invaded cells that crossed the inserts were stained with 0.1% crystal violet, and counted as the number of cells per field of view using phase-contrast microscopy.

### In situ hybridization (ISH) and data analyses

NKILA expressions in paraffin-embedded sections were examined using in situ hybridization (ISH) as previously reported [13].

### Electromobility shift assay (EMSA)

EMSA Assay was performed using LightShift Chemiluminescent EMSA Kit (20148, Pierce, Rockforld, IL) according to manufacturer's instructions. The sequence of canonical NFκB probe is AGTTGAGGGGACTTTCCCAGGC. The sequence of OCT1 EMSA probe as a loading control is TGTCGAATGCAAATCACTAGAA.

### Immunofluorescence

For immunofluorescent (IF) staining, the cells were incubated with primary antibodies against p65 (1:300, CST, USA), E-cadherin (1:400, CST, USA), vimentin (1:400, CST, USA), followed by incubation with Alexa Fluor 488/Alexa Fluor 555 secondary antibodies (1:1000, Life Technologies, USA). Then, the cells were counterstained with DAPI (Sigma, USA) and images were obtained by Confocal laser-scanning microscopy (Zeiss LSM710, Germany).

### Tumor xenografts

Female 4-6 weeks old NOD/SCID mice were used. All animal experiments were approved by Sun Yat-sen University laboratory animal care and use committee. 1×10^6^ Tscca and CAL27 cells with or without adjusting the expression of NKILA were injected in PBS intravenously in the mice tail vein. 8 weeks after tumor inoculation, the mice were sacrificed and livers and lungs were collected, weighed, fixed for paraffin-embedded section and RNA extraction. Paraffin section (4μm) were stained with hematoxylin and eosin (HE) [31].

### Statistical analyses

Statistical analyses were performed using SPSS version 16.0 (SPSS, Chicago, IL, USA). A Chi-squared test was used to analyze the relationship between NKILA expression levels and the clinicopathological characteristics. A one-way ANOVA was used to compare the NKILA expression levels between the TSCC tumors of different clinical stages. Survival curves were plotted using the Kaplan–Meier method and compared using the log-rank test. The survival data were evaluated using univariate and multivariate Cox regression analyses. Student's t-tests were used to calculate the p-value. P < 0.05 was considered significant. All experiments were performed at least 3 times and results were presented as mean±s.d.
